# Epigenetic Modification of TLRs in Leukocytes Is Associated with Increased Susceptibility to *Salmonella enteritidis* in Chickens

**DOI:** 10.1371/journal.pone.0033627

**Published:** 2012-03-16

**Authors:** Zhongyong Gou, Ranran Liu, Guiping Zhao, Maiqing Zheng, Peng Li, Huihua Wang, Yun Zhu, Jilan Chen, Jie Wen

**Affiliations:** Institute of Animal Science, Chinese Academy of Agricultural Sciences, Haidian, Beijing, China; Charité-University Medicine Berlin, Germany

## Abstract

Toll-like receptors (TLRs) signaling pathways are the first lines in defense against *Salmonella enteritidis* (*S. enteritidis*) infection but the molecular mechanism underlying susceptibility to *S. enteritidis* infection in chicken remains unclear. SPF chickens injected with *S. enteritidis* were partitioned into two groups, one consisted of those from Salmonella-susceptible chickens (died within 5 d after injection, n = 6), the other consisted of six Salmonella-resistant chickens that survived for 15 d after injection. The present study shows that the bacterial load in susceptible chickens was significantly higher than that in resistant chickens and *TLR4*, *TLR2-1* and *TLR21* expression was strongly diminished in the leukocytes of susceptible chickens compared with those of resistant chickens. The induction of expression of pro-inflammatory cytokine genes, *IL-6* and *IFN-β*, was greatly enhanced in the resistant but not in susceptible chickens. Contrasting with the reduced expression of TLR genes, those of the zinc finger protein 493 (*ZNF493*) gene and Toll-interacting protein (*TOLLIP*) gene were enhanced in the susceptible chickens. Finally, the expression of *TLR4* in peripheral blood mononuclear cells (PBMCs) infected *in vitro* with *S. enteritidis* increased significantly as a result of treatment with 5-Aza-2-deoxycytidine (5-Aza-dc) while either 5-Aza-dc or trichostatin A was effective in up-regulating the expression of *TLR21* and *TLR2-1*. DNA methylation, in the predicted promoter region of *TLR4* and *TLR21* genes, and an exonic CpG island of the *TLR2-1* gene was significantly higher in the susceptible chickens than in resistant chickens. Taken together, the results demonstrate that *ZNF493*-related epigenetic modification in leukocytes probably accounts for increased susceptibility to *S. enteritidis* in chickens by diminishing the expression and response of *TLR4*, *TLR21* and *TLR2-1*.

## Introduction

Toll-like receptors (TLRs) signaling pathways are the first lines in defense against Salmonella infection. The TLRs are broadly distributed on a variety of leukocytes [1], where they function as the primary sensors to initiate innate immune responses by responding to pathogen-associated molecular patterns (PAMPs) from bacteria, viruses, fungi or parasites [2], [3], [4]. The transcription factor NF-κB [5] is subsequently activated to induce the expression of immune and pro-inflammatory genes such as tumor necrosis factor alpha (*TNF-α*), interleukins 6, 1 beta, 8 and 12 (*IL-6*, *IL-1β*, *IL-8*, *IL-12*), and interferon (*IFN*), etc. [6], [7], [8].

Four TLRs (TLR4, TLR2, TLR9 and TLR5) are responsible for recognition of antigens from *S. enteritidis* in humans and mice. The dominant TLR involved in the host response to Salmonella infection is TLR4 [9]. Mutations in the *TLR4* gene increase the risk of Gram-negative infections in humans and mice [10], [11], [12] and mice deficient in both *TLR4* and *TLR2*, or *TLR4* and *TLR9*, were highly susceptible to *Salmonella typhimurium* [13]. Several specific avian TLR genes have been described. Avian *TLR2A* (*TLR2-1*) and *TLR2B* (*TLR2-2*) seem to have arisen from a duplication of *TLR2* found in other vertebrates [14] and avian *TLR21* is a functional homolog of mammalian *TLR9* [15].

Signaling pathways mediated by TLRs are tightly regulated to balance the activation and inhibition of inflammatory responses [16]. Multiple layers, involving many diverse factors, participate in negative regulation of TLR signaling. For example, suppressor of cytokine signaling 1 (SOCS1), phosphatidylinositol 3 kinase (PI3K), toll interacting protein (TOLLIP), and zinc finger protein A20 (A20) are intracellular negative regulators suppressing the signaling of TLR2, TLR4 and TLR9 in multiple pathways [17]. Transcriptional regulation of *TLRs* can also influence the inflammatory responses. In the clinical course of cystic fibrosis (CF), increased expression of *TLR2* caused chronic inflammation [18]. Diminished expression and function of *TLR1*, *TLR2* and *TLR4* accounts for T cell hyporesponsiveness in human filarial infection [19].

Little is known about the underlying mechanisms of transcriptional regulation of *TLRs* beyond *ZNF160*-dependent epigenetic regulation decreasing the expression of *TLR4* in intestinal epithelial cells [20], [21], [22]. While the *ZNF160* gene has not been identified in chicken, a Blastn search identified an avian homolog (*ZNF493*). Whether or not the same mechanism plays a role in modulating the immune response of the host to *S. enteritidis* infection remains unclear. Hypermethylation of promoter CpG dinucleotides has been associated with decreased expression of the gene [23], [24]. Some reports have indicated that methylation status of exonic CpG islands correlates with transcriptional activity [25]. In order to analyze the regulatory mechanism of TLRs, the methylation status in the promoter region and exonic CpG islands of TLRs were investigated.

Chickens are carriers of *S. enteritidis* that colonize the alimentary tract of chickens and, through excrement, can contaminate food products and water [26]. It was considered to be important to delineate part of the molecular mechanisms underlying differences in susceptibility of chickens to infection with *S. enteritidis*. The present study confirmed that the aberrant expression of *TLR4*, *TLR21*, and *TLR2-1* in peripheral blood leukocytes was associated with the susceptibility to *S. enteritidis* infection in chickens. More interestingly, it was demonstrated that the dysregulation of *TLR4*, *TLR21*, and *TLR2-1* was probably due to ZNF493-related epigenetic modification, including histone acetylation and DNA methylation.

## Results

### Increased bacterial load in susceptible chickens

The bacterial load in the blood at 0 h (before bacteria challenge), 8 h, 16 h, 24 h and 3 d post infection (TPI) were compared in six chickens that died within 5 d after infection with *S. enteritidis* (susceptible group) and six chickens that survived until 15 d TPI (resistant group). Results are presented in Table 1. *S. enteritidis* was not detected in any of the samples until 8 h TPI. From 16 h to 3 d TPI, the number of *S. enteritidis* in susceptible chickens was significantly higher (P<0.05) than that in resistant chickens. Notably, the bacterial load in susceptible chickens increased more dramatically at 16 h TPI and declined less significantly at 3 d TPI than that in resistant chickens. The results indicate that increased bacterial load is associated with susceptibility to *S. enteritidis* in chickens.

**Table 1:** Kinetics of *Salmonella Enteritidis* loads in inoculated SPF chickens determined by qPCR across all the times.

	0 h	8 h	16 h	24 h	3 d	12 d
S	0.00±0.00	6.54±0.32	7.05±0.23	6.96±0.06	6.87±0.21	
R	0.00±0.00	6.30±0.08	6.37±0.59	6.49±0.10	5.75±0.32	6.09±0.17
P value			P<0.05	P<0.05	P<0.05	

Data are presented as the mean bacterial loads and is expressed as log_10_ of the bacterial genome copy number per ml of blood (± SD) obtained from susceptible (S) and resistant (R) chickens.

### Decreased expression of *TLR4*, *TLR21* and *TLR2-1* genes in susceptible chickens

**Figure 1:**
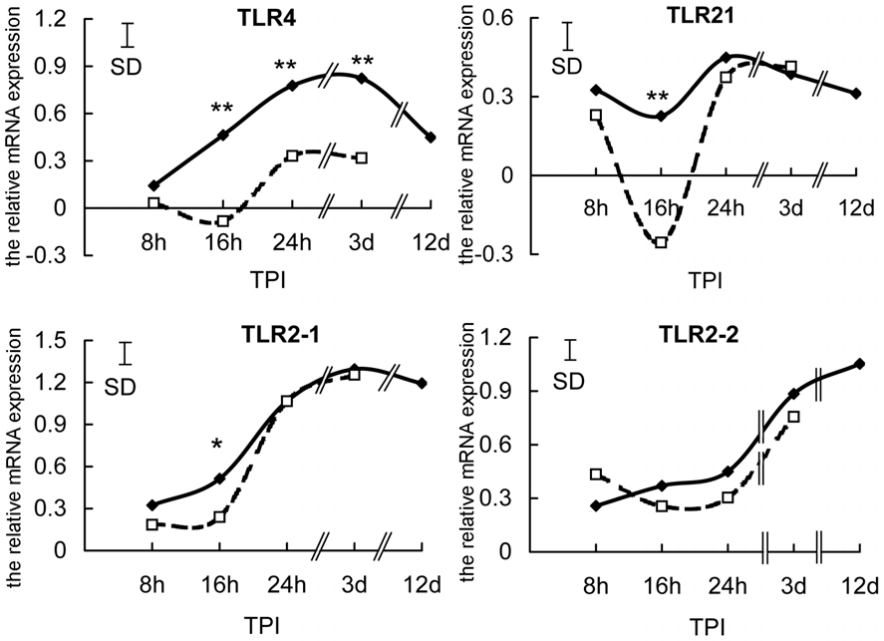
Decreased expression of *TLR4*, *TLR21* and *TLR2-1* genes in susceptible chickens. The relative expression of *TLR4*, *TLR21*, *TLR2-1* and *TLR2-2* in leukocytes of susceptible (□ ---- □) and resistant (⧫——⧫) chickens at 8 h, 16 h, 24 h, 3 d, and 12 d after infection with *S.enteritidis* is shown. Relative values, normalized using *β-actin* mRNA levels and the average expression levels in both groups at 0 h, are shown. The data are means (SD shown by the vertical bars) of 6 birds (*P<0.05; **P<0.01). TPI is time post-infection.

In order to explore the molecular mechanisms of susceptibility to *S. enteritidis* infection, the expression levels of Toll-like receptors (TLRs) were examined in susceptible chickens. The abundance of *TLR4*, *TLR21*, *TLR2-1* and *TLR2-2*, and transcripts and changes at all times post-inoculation were compared by q-RT-PCR in susceptible and resistant chickens. There were no significant differences in the expression of TLRs at 0 h (data not shown) and 8 h TPI between these two groups (Fig. 1), but, at later times, susceptible chickens had depressed expression of TLRs genes compared with resistant chickens. This was most evident at 16 h TPI, when *TLR4*, *TLR21* and *TLR2-1* transcripts were all significantly lower in the susceptible group than in the resistant group. Only *TLR2-2* mRNA did not differ between the two groups across all sampling times, whereas *TLR4* expression in resistant chickens was persistently and significantly higher than in susceptible chickens from 16 h to 3 d (Fig. 1). The results suggest that higher susceptibility to *S. enteritidis* and increased bacterial load might result from depressed expression of *TLR4*, *TLR21* and *TLR2-1* at the early stage of infection.

### Partially diminished inflammatory response in susceptible chickens

**Figure 2:**
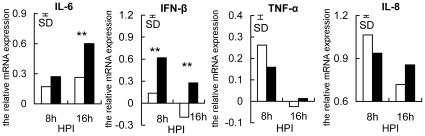
Partially diminished inflammatory response in susceptible chickens. The relative expression of *IL-6*, *IFN-β*, *TNF-α* and *IL-8* in susceptible (open bars) and resistant (filled bars) chickens at 8 h and 16 h after infection with *S.enteritidis* is shown. Data are means (n = 6), normalized to *β-actin* mRNA and the average expression at 0 h (**P<0.01). The vertical bar is the SD from the error mean square of the ANOVA. HPI is hours post-infection.

Four pro-inflammatory cytokine genes (*IL-6*, *IFN-β*, *TNF-α*, and *IL-8*) were used to investigate if the decreased expression of *TLR4*, *TLR21* and *TLR2-1* at 16 h in susceptible chickens resulted in a mitigated inflammatory response. Consistent with the expression of TLRs in the resistant and susceptible groups, the induction of *IL-6* and *IFN-β* transcription was greatly enhanced in the resistant, but not in the susceptible chickens at 16 h post-infection (Fig. 2). These results indicate that diminished expression of *TLR4*, *TLR21* and *TLR2-1* in the susceptible chickens leads to a decreased inflammatory response. The similar levels of *IL-8* in both groups demonstrated that only some of the pro-inflammatory cytokines showed down-regulation in susceptible chickens, perhaps because *IL-8* was regulated by other TLRs. In contrast, there was no obvious difference in the expression of *TNF*-*α* at 16 h, indicating that not all pro-inflammatory cytokine genes are induced at this early stage of infection (Fig. 2). Collectively, the results were consistent with higher susceptibility to *S. enteritidis* in birds being due to the partially diminished inflammatory response associated with decreased expression of *TLR4*, *TLR21* and *TLR2-1*.

### Enhanced expression of *TOLLIP* and *ZNF493* genes in susceptible chickens

**Figure 3:**
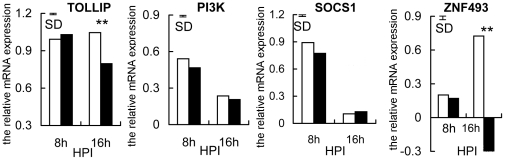
Enhanced expression of *TOLLIP* and *ZNF493* genes in susceptible chickens. The relative expression of *TOLLIP*, *PI3K*, *SOCS1* and *ZNF493* genes in susceptible (open bars) and resistant (filled bars) chickens at 8 h and 16 h after infection with *S. enteritidis* is shown. Data are means (n = 6), normalized to *β-actin* mRNA and the average expression at 0 h (**P<0.01). The vertical bar is the SD from the error mean square of the ANOVA. HPI is hours post-infection.

An attempt was then made to identify the molecular mechanisms responsible for decreased expression of *TLR4*, *TLR21* and *TLR2-1* in the susceptible chickens. The expression of four negative regulators of TLR2, TLR4 and TLR21 signaling pathways (*TOLLIP*, *PI3K*, *SOCS1* and *ZNF493*, a chicken homolog of mammalian *ZNF160*) was compared between susceptible and resistant chickens at 8 h and 16 h. There were no differences (P>0.05) between susceptible and resistant chickens in expression of *TOLLIP*, *PI3K*, *SOCS1* and *ZNF493* before infection or at 8 h TPI, when expression was increased in all birds. At 16 h, however, expression of *TOLLIP* and *ZNF493* in susceptible chickens was pronounced and exceeded that in the resistant chickens (P<0.01), while the other genes were up-regulated to lesser degrees and there were no differences between the two groups of chickens. Note the substantial increase in *ZNF493* transcripts between 8 h and 16 h in the susceptible chickens when those in resistant chickens changed in the opposite direction (Fig. 3).

### DNA methyltransferase inhibitor 5-Aza-dc and/or the histone deacetylase inhibitor TSA increased expression of *TLR4*, *TLR21* and *TLR2-1*

**Figure 4:**
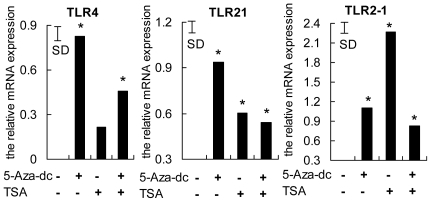
DNA methyltransferase inhibitor 5-Aza-dc and/or the histone deacetylase inhibitor TSA increased *TLR4*, *TLR21* and *TLR2-1* expression. Peripheral blood mononuclear cells were inoculated with *S. enteritidis* without additions (controls) or in the presence of 5-Aza-dc, TSA or TSA plus 5-Aza-dc. Relative abundances of *TLR4*, *TLR21* and *TLR2-1* mRNA were analyzed by qPCR and normalized to *β-actin* mRNA. Data are means (n = 3) and comparisons were made to expression in the controls (-,-). The vertical bar is the SD from the error mean square of the ANOVA, * indicates P<0.05.

The possibility that *TLR4*, *TLR21* and *TLR2-1* gene expression was regulated by epigenetic modification (histone acetylation and/or DNA methylation) in leukocytes infected with *S. enteritidis* was examined. Isolated PBMCs from SFP chickens were infected with *S. enteritidis* in the absence and presence of combinations of 5-Aza and TSA in the culture media. As shown in Fig. 4, 5-Aza-dc provoked a significant increase in *TLR4* expression. Either 5-Aza-dc or TSA was effective in up-regulating the expression of *TLR21* and *TLR2-1*; the effect of 5-Aza-dc was greater in the case of *TLR21* and that of TSA was greater for *TLR2-1*. No cooperative effects of 5-Aza-dc and TSA on the expression of the genes were observed. These results indicate that histone acetylation and DNA methylation are involved in the repression of *TLR4*, *TLR21* and *TLR2-1* expression in PBMCs of chickens during *S. enteritidis* infection.

### Higher methylation in the predicted promoter region of *TLR4* and *TLR21* gene, and an exonic CpG island of *TLR2-1* gene in susceptible chickens

**Figure 5:**
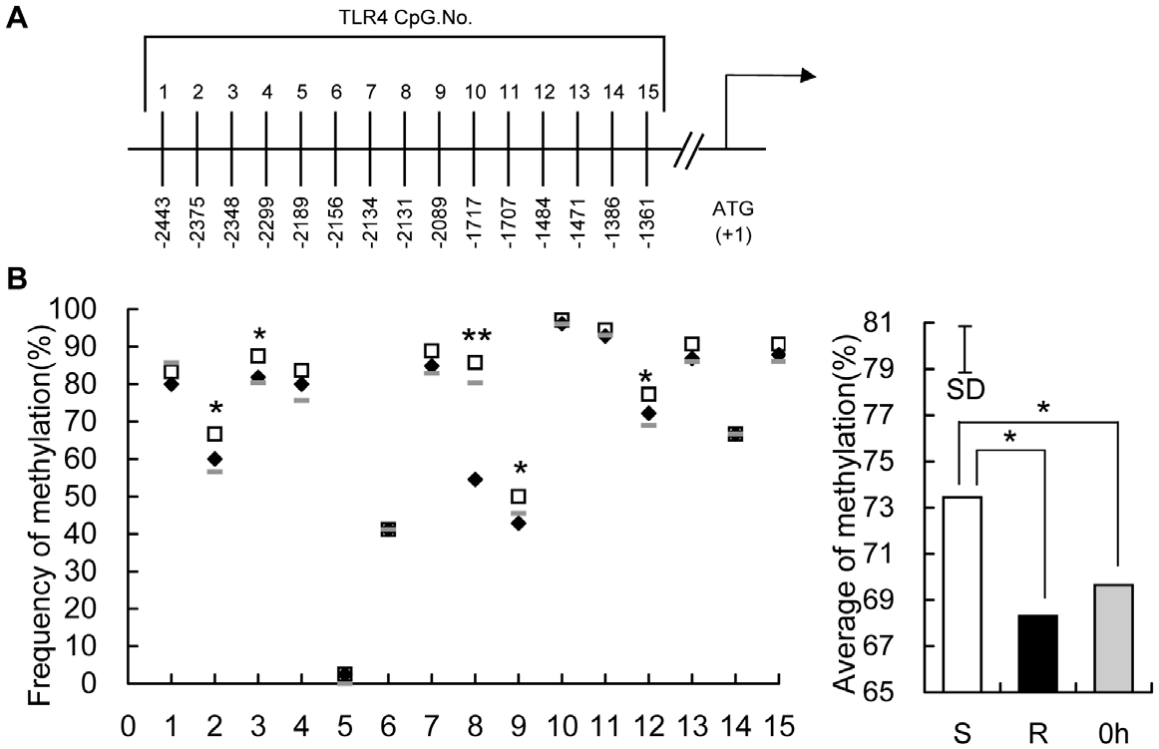
Methylation of 15 CpG motifs in the predicted promoter region of the *TLR4* gene. (A) The distribution of the 15 CpG dinucleotides from −2443 to −1361 in the upstream region of the *TLR4* gene relative to the translation start site (+1). (B) Genomic DNA from peripheral blood leukocytes of uninfected chickens at 0 h (⁃), susceptible (□) and resistant (⧫) chickens at 16 h TPI was modified with sodium bisulfite, amplified by PCR, cloned, and 12–16 independent clones were sequenced. The frequency of methylated CpGs in each CpG site (data are means of 12 birds for uninfected chicken and 6 birds for susceptible and resistant chickens, respectively) are shown and comparisons were made between susceptible and resistant chickens. The average of % methylation at each CpG site within all 15 CpGs in peripheral blood leukocytes of uncharged chickens (0 h, filled grey bars), susceptible (S, open bars) and resistant (R, filled black bars) chickens at 16 h after infection with *S. enteritidis* are presented. The vertical bar is the SD from the error mean square of the ANOVA, * indicates P<0.05, ** indicates P<0.01.

**Figure 6:**
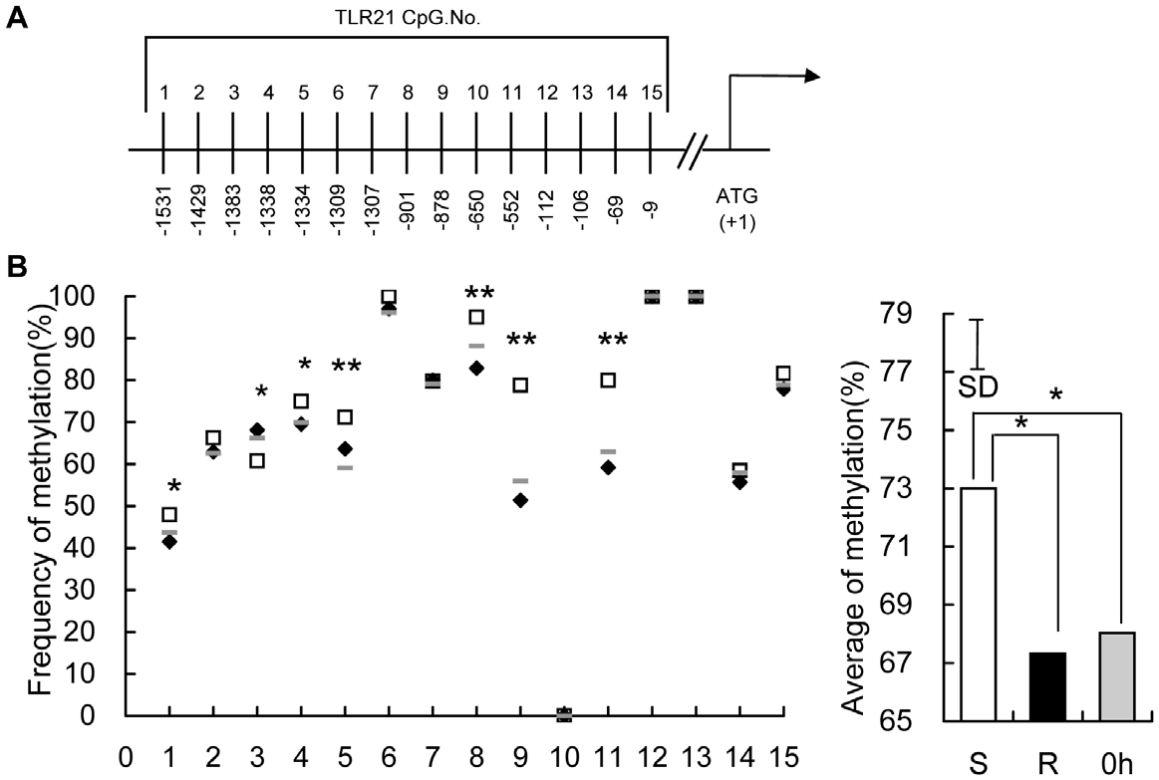
Methylation of 15 CpG motifs in the predicted promoter region of *TLR21* gene. (A) The distribution of the 15 CpG dinucleotides from −1531 to −9 in the upstream region of the *TLR21* gene relative to the translation start site (+1). (B) Genomic DNA from peripheral blood leukocytes of uninfected chickens at 0 h (⁃), susceptible (□) and resistant (⧫) chickens at 16 h TPI was modified with sodium bisulfite, amplified by PCR, cloned, and 12–16 independent clones were sequenced. The frequency of methylated CpGs in each CpG site (data are means of 12 birds for uninfected chicken and 6 birds for susceptible and resistant chickens, respectively) are shown and comparisons were made between susceptible and resistant chickens. The average of % methylation at each CpG site within all 15 CpGs in peripheral blood leukocytes of uncharged chickens (0 h, filled grey bars), susceptible (S, open bars) and resistant (R, filled black bars) chickens at 16 h after infection with *S. enteritidis* are presented. The vertical bar is the SD from the error mean square of the ANOVA, * indicates P<0.05, ** indicates P<0.01.

**Figure 7:**
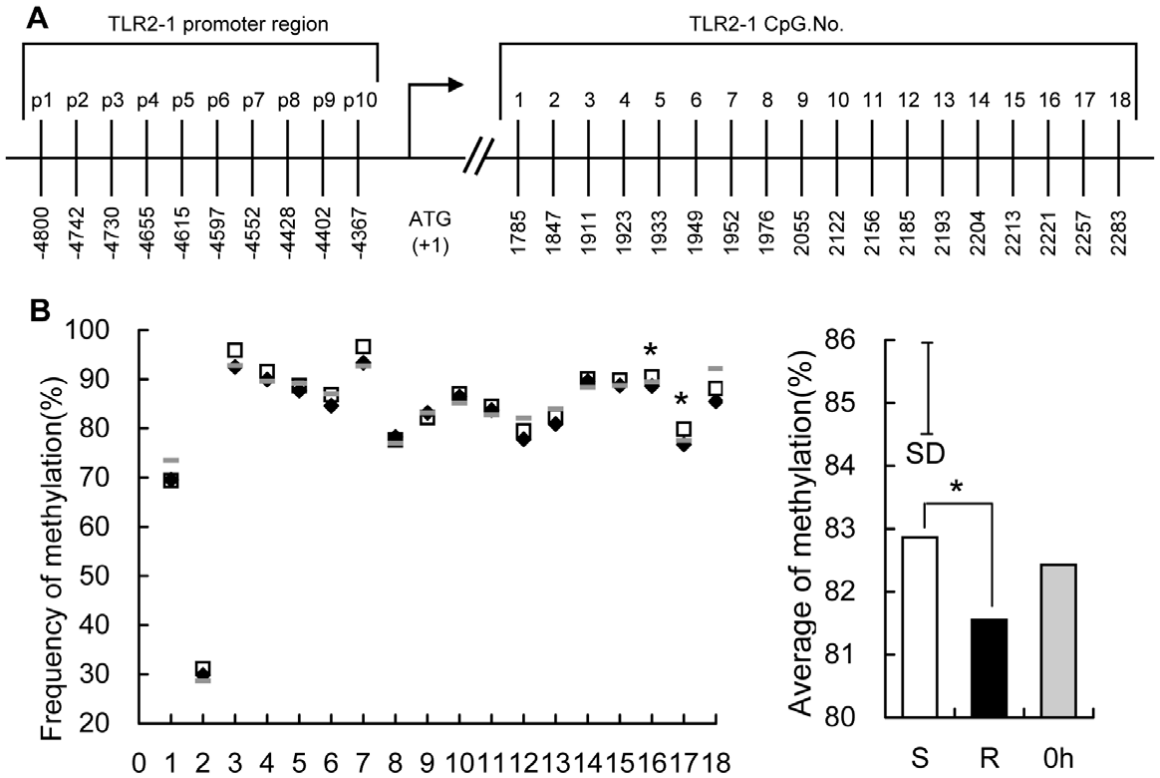
Methylation of 18 CpG motifs in the exon and 10 CpG motifs in the predicted promoter region of *TLR2-1* gene. (A) the distribution of the 18 CpG dinucleotides from 1785 to 2283 in the exon region and 10 CpG dinucleotides from −4800 to −4367 in the predicted promoter region of the *TLR2-1* gene relative to the translation start site (+1). (B) Genomic DNA from peripheral blood leukocytes of uninfected chickens at 0 h (⁃), susceptible (□) and resistant (⧫) chickens at 16 h TPI was modified with sodium bisulfite, amplified by PCR, cloned, and 12–16 independent clones were sequenced. The frequency of methylated CpGs in each CpG site (data are means of 12 birds for uninfected chicken and 6 birds for susceptible and resistant chickens, respectively) are shown and comparisons were made between susceptible and resistant chickens. The average of % methylation at each CpG site within all 18 CpGs in peripheral blood leukocytes of uncharged chickens (0 h, filled grey bars), susceptible (S, open bars) and resistant (R, filled black bars) chickens at 16 h after infection with *S. enteritidis* are presented. The vertical bar is the SD from the error mean square of the ANOVA, * indicates P<0.05.

The possibility that diminished expression of *TLR4*, *TLR21* and *TLR2-1* at 16 h TPI might be due to differences in methylation was explored at multiple locations within each of these genes, using leukocyte DNA at 0 h and 16 h TPI. For both *TLR4* (Fig. 5A) and *TLR21* (Fig. 6A), 15 CpG motifs in the predicted promoter regions were assessed. In the case of *TLR2-1*, 10 CpG motifs within the promoter and 18 CpG motifs in an exonic CpG island (Fig. 7A) were evaluated. There were no differences (P>0.05) in the methylation of *TLR4*, *TLR21* and *TLR2-1* genes between the susceptible and resistant chickens at 0 h (data not shown), and the average methylation level of all the 12 chickens before infection is shown as the basic methylation status. Interestingly, the average methylation levels of *TLR4* and *TLR21* at 16 h rose dramatically from the basic level at 0 h in susceptible chickens whereas it fell slightly (around 1%) in resistant chickens. Thus, higher methylation in the predicted promoter region of the *TLR4* and *TLR21* genes, was evident in susceptible versus resistant chickens at 16 h (Fig. 5B, Fig. 6B). This trend was also evident in several CpG sites (5 sites for *TLR4*, 7 for *TLR21*). No significant differences were observed in the promoter region of the *TLR2-1* gene between these two groups at 16 h (data not shown), but an exonic CpG island of the *TLR2-1* gene showed higher methylation level in susceptible than resistant chickens, although the difference was not as great as that in *TLR4* and *TLR21* (Fig. 7B).

Collectively, the results presented here show that diminished expression and response of *TLR4, TLR21 and TLR2-1* in peripheral blood leukocytes, due to epigenetic modification, likely account for increased susceptibility to *S. enteritidis* in chickens.

## Discussion

Although the physiological importance of transcriptional regulation of *TLRs* is unclear, several reports indicate that it directly influences the immune response of the host. The expression of *TLRs*, specifically *TLR2* and *TLR4*, is induced by various PAMPs from bacteria, viruses, fungi or parasites for inflammatory responses in macrophages, epithelia, cecum and spleen [27], [28], [29]. Dysregulated expression of *TLRs* can impair the immune response of the host, resulting in various diseases. In the clinical course of cystic fibrosis (CF), dysregulated expression of *TLR2* caused chronic inflammation [18]. Diminished expression and function of *TLR1*, *TLR2* and *TLR4* accounts for T cell hyporesponsiveness in human filarial infection [19]. The fact that various expression patterns of TLRs appear in tissues with different immune responses and function demonstrates the important role of transcriptional regulation of TLRs in the signaling of TLRs. For example, in enterocytes, depressed expression of *TLR4* contributes to maintenance of intestinal homeostasis [22]. The downregulation of *TLR5* expression was observed in cecum by *S. enteritidis* infection, which might be beneficial to protect host cells from overstimulation by bacterial flagellin [29]. In addition, genetic line has significant effect on TLR expression, which may partly explain genetic variability in immune response to *S. enteritidis* [30]. In this study, the reduced expression of the pro-inflammatory cytokines *TNF-α* and *IL-6* in leukocytes of susceptible chickens (Fig. 2), confirmed that reduced expression of *TLR4*, *TLR21* and *TLR2-1* constrained the immune response to *S. enteritidis*, which is consistent with the human studies.

While not previously described for *S. enteritidis* infection, epigenetic regulation of *TLR4* and *TLR21* involving ZNF493 in chickens, participates in the negative regulation of *TLRs*. The avian ZNF493 examined here (and the mammalian homolog ZNF160) are Kruppel-related zinc finger proteins with an N-terminal repressor domain, the Kruppel associated box (KRAB), a potent repressor of transcription [31]. The mechanism involves recruiting KRAB-associated protein 1 (KAP1), triggering *de novo* DNA methylation [32], and the forming of a multimolecular complex comprising histone deacetylases, which induces transcriptional repression through the formation of heterochromatin [33], [34], [35]. The present study shows a dramatic enhancement of *ZNF493* expression in susceptible chickens at 16 h, contrasting with diminished expression of *TLR4* and *TLR21* (Fig. 3). This finding prompted the experiment using chicken PBMCs infected with *S. enteritidis in vitro*, which demonstrated that expression of *TLR4* and *TLR21* was significantly promoted by inhibitors of DNA methyltransferase and histone deacetylase (Fig. 4). In addition, the susceptible, but not the resistant, chickens had increased methylation of *TLR4* and *TLR21* genes at 16 h compared with their basal levels at 0 h, which is consistent with the increased expression of *ZNF493* in the susceptible chickens at 16 h (Fig. 5, Fig. 6). All of these findings indicate that ZNF493-related epigenetic regulation of *TLR4* and *TLR21* in leukocytes plays a role in the negative regulation of *TLRs* in chickens. Two possibilities might explain the differences at the transcriptional level of *ZNF493* gene in susceptible and resistant chickens: (1) polymorphisms in the regulatory regions, including promoter of the *ZNF493* gene; (2) polymorphisms of regulatory genes of the *ZNF493* gene. White Leghorn chickens are known to have genetic variability in resistance to *S. enteritidis* among different strains [36]. The SPF chicken used in the present study is a commercial Babcock® White Leghorn line, which is very likely to have multiple genetic origins and genetic variability in susceptibility to *S. enteritidis*. The present study, however, shows that no polymorphisms in the promoter region of avian *ZNF493* gene were detected in susceptible and resistant chickens (data not shown). It implies that diminished expression of *ZNF493* gene might result from the polymorphisms of its regulatory genes or other regulatory regions (introns, 3′-UTR…).

There is little known about the overall transcriptional regulatory mechanism of *TLRs*. Based on the known reports, it can be inferred that positive transcriptional regulation of TLR by cytokines to augment TLR signaling and negative feedback control from negative regulators to terminate activation of TLRs are the basic mechanisms of transcriptional regulation of TLRs [17], [37], [38]. Moreover, transcriptional regulation of *TLRs* varies in different tissues, indicating that tissue-specific genes modify the regulatory system [22], [29]. In addition, the pathogen probably can also exploit and modulate the regulatory system, disturbing the normal expression of *TLRs* [18], [19], [39], [40]. In the present study, the expression of TLRs showed a common trend in obviously rising to the maximal level at around 3 d, followed by a fall by 12 d (Fig. 1). This trend indicates positive regulation by cytokines played a role in the initial upregulation stage and negative feedback control in the later downregulation stage. The epigenetic modification of TLRs in this study seems to be driven by two opposite mechanism, methylation and demethylation, depending on the particular TLR. *TLR4* and *TLR21*, but not TLR2-1 showed an obvious downregulation and increase in methylation level in susceptible chickens at 16 h TPI, which probably resulted from ZNF493-related negative epigenetic modification. For all the three genes, the abundance of mRNA increased significantly compared with that at 0 h and the methylation level in resistant chickens similarly declined slightly from the basic level (Fig. 5, Fig. 6, Fig. 7), indicating that demethylation was widely involved in the regulation of *TLRs*. This demethylation happened in resistant chickens with higher expression of inflammatory proinflammatory cytokines, indicating that it could be one of the positive regulatory mechanisms of the cytokines.

The role for ZNF493-related epigenetic regulation of TLRs in the response to infection with *S. enteritidis*, however, seems to be quite different from the basic negative regulatory mechanism of the TLR signaling pathway. Immune signaling pathways mediated by TLRs are tightly regulated to avoid over-activation of inflammatory responses and most negative regulators use a mode of negative feedback to terminate *TLRs* activation. They are induced by the activation of *TLRs*, or are constitutively expressed, but could possibly exert their functions only when *TLRs* are over-activated [41], [42]. Since the diminished expression of *TLRs* and induction of *ZNF493* in the susceptible chickens occurred at the early stage of *S. enteritidis* invasion when induction of inflammatory response genes was even lower than in the resistant chickens (Fig. 2), and expression of *TLR4*, *TLR21* and *TLR2-1* remained at low levels (Fig. 1), it is not reasonable to account for the induction of *ZNF493* by negative feedback control from the host and, instead, it might have been provoked by *S. enteritidis*. *S. enteritidis* almost certainly benefits from the diminished expression of *TLR4*, *TLR21* and *TLR2-1* for its successful invasion and colonization of the susceptible host. Indeed, *S. enteritidis* secretes virulence factors to temporarily turn off TLR signaling to aid in colonization of host cells by inactivating IRAK, a kinase in the signaling pathway [39], [40].

All of the findings described here, comparing blood bacterial load, transcript abundance and DNA methylation in leukocytes of susceptible and resistant chickens, along with the effects of inhibitors for epigenetic modification on transcript abundance in isolated PBMCs, are consistent with *S. enteritidis* being able to provoke epigenetic regulation of the transcription of *TLR4*, *TLR21* and *TLR2-1* as an important strategy for weakening host defenses.

## Materials and Methods

### Bacterial Strains and Infections

*S. enteritidis* (50041) was obtained from the China Institute of Veterinary Drugs Control (IVDC, Beijing, China) and was used for all infections. Bacteria were resuscitated overnight in Luria–Bertani (LB) broth at 37°C in an orbital shaking incubator at 150 rpm. The number of CFU of *S. enteritidis* was determined by plating serial dilutions.

### SPF Chickens and *In Vivo* Infections

Animal studies were performed according to protocols approved by the Beijing Laboratory Animal Use and Care office (approval number: SYXK 2006-0027). Specific-pathogen-free White Leghorn chickens were purchased from the Beijing Laboratory Animal Research Center (BLARC, Beijing, China). Birds were reared in separate cages in the SPF chicken experimental center of Beijing Academy of Agriculture and Forestry Sciences (Beijing, China) and given *ad libitum* access to water and a diet specifically designed for SPF chickens (BLARC). Birds were confirmed to be free of Salmonella by culturing faecal samples in buffered peptone water (BPW) overnight with shaking at 150 rpm followed by spreading and culture (37°C, 18–24 h) on brilliant green agar containing 100 mg/ml nalidixic acid [43].

Chickens (n = 20) aged 30 d, were blood sampled (0 h) then injected intramuscularly into the breast with 0.5 ml PBS containing 8.7×10^8^ CFU *S. enteritidis* (50041) and additional blood samples were taken at 8 h, 16 h, 24 h, 3 d, and 12 d. Blood from the wing vein (0.5 ml) was taken into EDTA-coated syringes and held on ice for ∼1 h before lysing and isolating leukocytes (see below). Nine chickens died within 5 days, 4 died between the 5th and the 8th day after injection and the remaining 7 survived until 15 d. Before detailed analyses were performed, the chickens and their samples were partitioned into two groups, one consisted of those from Salmonella-susceptible chickens (died within 5 d after injection, n = 6), the other consisted of six of the total of seven Salmonella-resistant chickens that survived for 15 d.

### Quantitative RT-PCR (qPCR)

Erythrocytes in blood samples were lysed with Red Blood Cell Lysis Buffer (Roche, Shanghai, China) to isolate peripheral blood leukocytes. Total RNA from leukocytes or cultured PBMCs was prepared using Trizol reagent (Invitrogen, USA) and purified with an RNA cleaning kit (Tiangen, Beijing, China) after treatment with RNase-free DNase to eliminate any gDNA contamination. Total RNA was quantified with a NanoDrop 2000 Spectrophotometer (Thermo Scientific, USA) and formaldehyde gel electrophoresis, and adjusted to the 500 ng/µl. First-strand cDNA was synthesized from 2 µg total RNA (Promega, Beijing, China). Specific mRNAs were quantified by qPCR with an ABI 7500 Real-time Detection System (Applied Biosystems, USA) using a SYBR® Premix Ex Taq™ II kit (Takara, Dalian, China); the primers used (Beijing Genome Institute, Beijing, China), based on chicken sequences, were designed by Primer Premier 5.0 and are listed in Table 2. The amplification was performed in a total volume of 20 µl, containing 10 µl of 2× SYBR Green I real-time PCR Master Mix (ABI), 0.4 µl ROX, 2 µl of the 3×diluted cDNA, 1 µl of each primer(10 µmol), and 5.6 µl ddH_2_O. The concentrations of primers and cDNA were optimized to ensure similar PCR efficiencies (close to 100%) between the target genes and the reference gene (*β-actin*), if needed. The real-time PCR program started with denaturing at 95°C for 1 min, followed by 40 cycles of 95°C for 15 s and 60°C for 60 s. Dissociation analysis of amplification products was performed after each PCR to confirm that only one PCR product was amplified and detected. Data were analyzed with ABI 7500 SDS software (ABI) with the baseline being set automatically by the software and values of average dCT (normalized using *β-actin*) was exported into Excel for the calculation of relative mRNA expression. The comparative CT method was used [44], to determine fold-changes in gene expression, calculated as 2^−▵▵CT^ using average expression levels in samples of both groups at 0(h as the calibrator (assigned an expression level of 0). Results were expressed as relative mRNA expression which was log(2^−▵▵CT^) at each time, from triplicate analyses.

**Table 2:** qPCR primers used in this study.

Gene name	Sequence	GenBank No.
*β-actin*	f-5′-GAGAAATTGTGCGTGACATCA-3′	L08165
	r-5′-CCTGAACCTCTCATTGCCA-3′	
*TLR4*	f-5′-AGTCTGAAATTGCTGAGCTCAAAT-3′	AY064697
	r-5′-GCGACGTTAAGCCATGGAAG-3′	
*TLR2-1*	f-5′-TTACCGGTGCTTCATTCACA-3′	NM_204278
	r-5′-CATATCCCATGCTCCTTTCC-3′	
*TLR2-2*	f-5′-TGTACACTCTTGGGCACTGG-3′	NM_001161650
	r-5′-CATGGCACCAGAAACACCTT-3′	
*TLR21*	f-5′-GATGGAGACAGCGGAGAA-3′	NM_001030558
	r-5′-GCGGAAGTACAAAGGTGC-3′	
*TNF-α*	f-5′-GAAGCAGCGTTTGGGAGT-3′	AY765397
	r-5′-GTTGTGGGACAGGGTAGG-3′	
*IL-8*	f-5′-AACAAGCCAAACACTCCT-3′	NM_205498
	r-5′-AGGGTGGATGAACTTAGAAT-3′	
*IL-6*	f-5′-GCAGGACGAGATGTGCAA-3′	NM_204628
	r-5′-CCAGGTAGGTCTGAAAGGC-3′	
*IFN-β*	f-5′-CCAGCTCCTTCAGAATACG-3′	NM_001024836
	r-5′-TGCGGTCAATCCAGTGTT-3′	
*PI3K*	f-5′-AACATCTGGCAAAACCAAGG-3′	NM_001004410
	r-5′-CTGCAATGCTCCCTTTAAGC-3′	
*SOCS1*	f-5′-GCCCATGAGAAGCTGAAGTC-3′	NM_001137648.1
	r-5′-GGGGTGACCAATACCTTCCT-3′	
*TOLLIP*	f-5′-AAGGCAGGGTGATGACAAAG-3′	NM_001006471
	r-5′-AGGAGGTGGTATTGCCACAG-3′	
*A20*	f-5′-GACAGGCTGATGCAACTTGA-3′	XR_026935
	r-5′-CAAACCCAGAACCGTTCACT-3′	
*ZNF493*	f-5′-CGGAGCACAACGACTGTAGA-3′	XM_001236375
	r-5′-GAGAAGCACAGGGGTTGAAG-3′	

### Determination of bacterial load in blood

The bacterial load in the blood of chicken was estimated by serovar-specific qPCR assay as described previously [45], [46]. The probe (5′-FAM-TGCAGCGAGCATGTTCTGGAAAGC-TAMRA-3′) and primers set (the forward primer, 5′-TCCCTGAATCTGAGAAAGAAAAACTC-3′; the reverse primer, 5′-TTGATGTGGTTGGTTCGTCACT-3′) were designed from the SdfI gene (Gen-Bank Accession No. AF370707.1). Bacterial DNA isolated from peripheral blood of chickens at 0h, 8h, 16h, 24h, 3d, and 12d was amplified using a real-time PCR core kit (R-PCR version 2.1, Takara, Dalian, China) in a 25µL reaction mixture containing 0.6µL of each primer (10µmol/L), 0.75µL of deoxyribonucleotide triphosphates (10mmol/L), 1.25 U of Ex Taq DNA Polymerase (Ex Taq Hot Start Version, Takara), 5µL of 5× PCR buffer (Mg2+ free), 0.8µL of TaqMan probe (5µmol/L), 0.5µL of Mg2+ (250mmol/L), and 5µL of templates. Each PCR run consisted of a 5(min hot start at 95°C, followed by 40 cycles consisting of 30 s of denaturation at 94°C, 30 s of annealing at 55°C, and a fluorescence read step. *S. enteritidis* DNA was isolated from bacterial cultures and CFU of *S. enteritidis* in the bacterial cultures was quantified by serially dilutions in BPW and plating, as the number of genomic copies. To extrapolate the bacterial number in each blood sample, serial dilutions of the genomic DNA were amplified (copy number ranging from 10^2^ to 10^8^).

### Isolation and culture of peripheral blood mononuclear cells (PBMCs)

Peripheral blood mononuclear cells (PBMCs) were isolated from a separate group of six 30 d-old SPF chickens using Ficoll-Hypaque, specific gravity 1.077 (Tian Jin Hao Yang Biological Manufacturing Co., Tianjin, China). Briefly, fresh, non-coagulated blood, diluted 1∶1 in Ca^++^, Mg^++^-free Hanks' balanced salt solution (Sigma, Shanghai) was overlaid and centrifuged at 1500 rpm for 30 min. to obtain the 1.077 band. The PBMCs were collected and washed twice in RPMI 1640 medium (Invitrogen, USA) and resuspended in fresh RPMI 1640. The cell concentration was adjusted to 1.5×10^7^ PBMCs/ml and 2 ml were cultured in 1640 medium containing 10% (v/v) fetal bovine serum (Biowest; Beijing, China). Cells were cultured at 37°C in a humidified incubator under 5% CO_2_.

### Inhibition of histone deacetylase and DNA methyltransferase in PBMCs

Cells, prepared as above, were inoculated with 1×10^5^ CFU *S. enteritis* in PBS and treated as follows: 10 µM 5-aza-2-deoxycytidine (5-Aza-dc; Sigma, Shanghai, China) for 3 d; 80 nM trichostatin A (TSA; Shanghai, Sigma) for 24 h, or with TSA plus 5-Aza-dc for an additional 24 h after initial treatment with just 5-Aza-dc for 2 d. Cells were then harvested to obtain total RNA. Transcripts of *TLR4*, *TLR21*, and *TLR2-1* were quantified by qPCR as described below.

### Bisulfite conversion reaction and DNA sequencing

Genomic DNA from peripheral blood leukocytes of susceptible (n = 6) and resistant (n = 6) chickens at 16 h after infection with *S. enteritidis* was prepared using the phenol/chloroform method. To analyze methylation of CpG motifs, 500 ng of genomic DNA was denatured at 98°C for 10 min, modified by the conversion reagent (bisulfite) at 64°C for 2.5 h, and then purified using an EZ DNA Methylation-Gold Kit™ (Zymo Research, Beijing, China).

The promoter region (including core promoter, proximal promoter and distal promoter) of the *TLR4* and *TLR21* genes were amplified by PCR from the sulfite-modified genomic DNA using two pairs of primers of *TLR4* (TLR4-P1, TLR4-P2) and *TLR21* (TLR21-P1, TLR21-P2, TLR21-P3). The promoter region and a predicted CpG island in the exon of *TLR2-1* were amplified using PCR primer pairs TLR2-1-P1 and TLR2-1-P2 (Table 3). CpG islands were found (http://www.uscnorris.com/cpgislands2/cpg.aspx) using 50% GC; ObsCpG/ExpCpG, 0.60; length, 300 bp; and gap between adjacent islands, 100 bp. PCR amplifications were performed using the GoTaq® Hot Start Colorless Master Mix (Promega). Following purification of PCR products, they were cloned into the pMD-18T vector for sequencing; 12–16 clones from each sample were analyzed.

**Table 3:** Primers for methylation detection.

Primer name	Sequence	Gene ID
TLR4-P1	f-5′-AAAAGTAGATTGATTTTTAATGTGGA-3′	417241
	r-5′-TGTTTTTTTTTGTAGA GTTTAGG-3′	
TLR4-P2	f-5′-AGAGATTTTTGATGATTTTATTAGA-3′	417241
	r-5′-GTAATTGTAAAGTTATTTTTGGG-3′	
TLR21-P1	f-5′-GTTGTTAGTAGATATTTTTTGGTAGG-3′	415623
	r-5′-AATATCTAATTCCCTTCATCAATAA-3′	
TLR21-P2	f-5′-TTATTTAGTGGGTAGTGGGGTT-3′	415623
	r-5′-AACAAAACTAAAAAAAACCAATAA-3′	
TLR21-P3	f-5′-TAGAGTATTAGGGAGGTGGTATAG-3′	415623
	r-5′ACTCAATAACACCATCCCAATA-3′	
TLR2-1-P1	f-5′-AAATTTTGTTTTTAGATTTGTGATT-3′	374141
	r-5′-CTACAACCCTCTCATCCTACCA-3′	
TLR2-1-P2	f-5′-AGGTTGGGAGTGTGTAGTTGTTAT-3′	374141
	r-5′-AGAAGTAGTTTTTTGGTGAGGT-3′	

### Statistical analysis

When needed for normality and homogeneity of variance, data were log-transformed. Analyses were by two-way GLM ANOVA (*in vivo* study) or one-way (*in vitro* study) ANOVA using SAS (version 8.0). The models were:
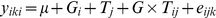
(1)

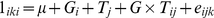
(2)

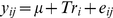
(3)

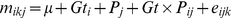
(4)where: y = relative mRNA expression (log-transformed); l = bacterial load; m = the frequency of methylated CpG; μ = population mean; G = the effect of 2 different groups (resistant and susceptible chickens); T = the effect of time (8 h, 16 h, 24 h, 3 d or 12 d after infection); Tr = the effect of 4 treatment combinations (5-Aza-dc, TSA: −,−; +,−; −,+; +,+); Gt = the effect of three different groups (uninfected, resistant and susceptible chickens); P = the effect of CpG positions (15 CpG positions for TLR4 and 18 for TLR2-1) and e was the random error. Multiple comparisons of means were performed using Duncan's multiple range tests and the SD derived from the Error Mean Squares. Significance was set at p<0.05 (highly significant if p<0.01).
